# Status and Perspectives of Clinical Modes in Surgical Patients With Lung Cancer: A Retrospective Study

**DOI:** 10.1097/MD.0000000000002429

**Published:** 2016-01-15

**Authors:** Yutian Lai, Heng Du, Xin Wang, Cheng Shen, Jian Huang, Weiming Li, Guowei Che

**Affiliations:** From the Department of Thoracic Surgery, West China Hospital, Sichuan University, Chengdu, P.R. China (YL, HD, XW, CS, JH, GC); and Department of Respiratory Medicine, West China Hospital, Sichuan University, Chengdu, P.R. China (WL).

## Abstract

To investigate the association between the clinical characteristics and clinical modes of surgically treated lung cancer patients, we conducted a retrospective study with 1097 lung cancer patients receiving pulmonary resection between 2012 and 2013.

A physical examination or screening (PES) group (n = 267) and a symptomatic (SY) group (n = 830) were established depending on the new clinical mode (sequence of physical examination, early detection and sequential medical treatment) and the conventional mode (hospitalization due to occurrence of relevant symptoms), respectively.

A higher proportion of patients referred to our unit directly form a junior medical unit is found in PES group (43.8%, 117/267 vs 13.6%, 113/830) (*P* < 0.001) and 37.5% (100/267) patients in PES group spent <1 months from detection or first medical visit to diagnosis compared with 15.4% (128/830) patient in SY group (*P* < 0.001). A significantly higher proportion of PES patients versus SY patients received video-assisted thoracoscopic surgery (VATS) resection (67.8%, 183/267 vs 42.6%, 352/830; *P* < 0.001). A significantly higher proportion of PES patients versus SY patients chose sublobar resection (16.9%, 45/267 vs 7.6%, 63/830; *P* < 0.001). A significantly higher proportion of PES patients versus SY patients are at stage 0 or I (64.4%, 172/267 vs 40.7%, 338/830; *P* < 0.000). The postoperative incidence rate of complications in 30 days is significantly higher in SY group than in PES group (34.9% vs 27.3%; *P* = 0.022).

Helping to early diagnosis and surgical treatment, early tumor detection via PES may contribute to significantly higher proportions of early-stage lung cancer, use of VATS pulmonary resection, and sublobectomy as well as lower complication rate.

## INTRODUCTION

Malignant tumors are the most health-threatening and death-causing disease to humans. Among all tumors, the morbidity rate of lung cancer ranks first in China since about 600,000 people were attacked in 2010 and 480,000 of them died.^1,2^ Despite advances in treatment, lung cancer remains largely incurable because most cancers are diagnosed at the advanced stage, rather than the intermediate stage when the pre-malignant or early lesions are amenable to resection and cure. As early-stage lung cancer can be most effectively treated by lobectomy, early diagnosis and treatment planning may be an effective means to decrease the mortality rate of lung cancer. However, diagnosis is often achieved at such late stage that the chance of radical surgery is lost in about two-thirds of patients. There are few ideal methods for early lung cancer diagnosis because of the heterogeneity of clinical manifestations and pathologic characteristics. Several strategies aim to detect lung lesions at an earlier stage, such as screening imaging methods (eg, chest x-ray or chest computed tomography), and screening with biomarkers in blood, which are involved in regular programs of periodic physical examination or screening (PES) in many relevant organizations. Fortunately, periodic PES becomes available to more people through the improvement of public health awareness and social medical insurance, the evolution of social security systems, and the development of PES organizations. The prevalence of PES increases the possibility of lung cancer detection and impels patients, especially asymptomatic patients, to make a medical visit. The new clinical mode in the sequence of physical examination, early detection, and sequential medical treatment results in significantly different characteristics in clinic, compared with the conventional mode in which hospitalization is due to the occurrence of relevant symptoms. However, the data and analyses concerned are insufficient. We therefore perform a comparative analysis to investigate the status and perspectives of clinical modes in surgical patients with lung cancer and confirm this conclusion. This study may help with policy-making in clinic and public health.

## MATERIALS AND METHODS

### Study Population

From a total of 1455 lung cancer patients identified in our unit, we excluded patients who finally did not accept surgical treatment due to various reasons, had secondary lung cancer, had histology of cancers other than nonsmall cell lung cancer (NSCLC), or had incompleteness or loss of relevant data. Finally, a total of 1097 lung cancer patients undergoing surgical pulmonary resection in our unit between January 2012 and December 2013 were included. The demographic, clinical, and surgical data of all the included patients were retrospectively analyzed. The pathological stages were determined using the tumor node metastasis (TNM) Staging System edition 7 from the Union for International Cancer Control (UICC).^3,4^

### Grouping Criterion

The 1097 patients following the clinical mode or the conventional mode were divided into a PES group and a symptomatic (SY) group, respectively. Patients following the new clinical mode were ascertained by relevant reports or documents offered by PES organizations or hospitals and relevant PES records or data that could be found in relevant electronic systems of institutions.

### General Introduction of Medical Insurance Framework of China

Universal health coverage has been promoted by the administration of China, with remarkable achievements in health care reform. Basically, the basic medical insurance system of China is composed of new cooperative medical systems mainly aiming at people in rural regions and urban social medical system for urban residents, according to various factors of the status of China.^5,6^ Differences in medical systems, distribution of medical resources as well as economic development may influence the clinical modes of lung cancer patients in urban or rural regions. Therefore, we also analyzed the distributive differences of the clinical modes between urban or rural areas.

### Surgical Approaches and Types of Resection

Lobectomy is regarded as the standard surgical mode for lung cancer treatment. We consider that sublobectomy, especially segmentectomy, could be 1 standard surgical mode for treatment of early-stage lung cancer, and produce similar survival as lobectomy for stage 0 or I patients with tumor smaller than 2 cm in size. Sublobectomy was recommended to those patients mainly for better preservation of lung function.^7,8^

Video-assisted thoracoscopic surgery (VATS) lobectomy has been considered as an optional approach for surgical lung cancer patients, especially for peripheral NSCLC with the size smaller than 5 cm, or minimally invasive central NSCLC, considering the advantages of alleviated postoperative pains, shorter in-hospital duration, and better preservation of lung function. Also, patients’ opinions, feasibility, and availability of surgical approaches should be adequately considered.

### Statistical Analysis

SPSS 19.0 and Stata 12.0 (Stata Corp, College Station, TX) were used for statistical analysis. All statistical tests were 2-sided. Data were presented as means ± standard deviations (mean ± SD) for continuous variables. Discrete variables were analyzed using the Chi-square test or Fisher exact test. Distribution of time durations from detection or the first medical visit to diagnosis and pathway of being admitted in our unit between the 2 groups were analyzed by nonparametric test (Wilcoxon rank sum test). All results were considered significant at *P* <0.05.

### Ethical Review

This study is an observational and retrospective study, approved by the Regional Ethics Committee of West China Hospital of Sichuan University.

## RESULTS

Totally, 1097 patients (mean age, 58.8 ± 10.0 years; age range, 15–86 years) were involved in the population analysis, including 707 (64.4%) men and 390 (35.6%) women. There were 413 (37.6%) smokers, 177 (16.1%) ever-smokers, and 507 (46.2%) nonsmokers (Table 1).

**TABLE 1:**
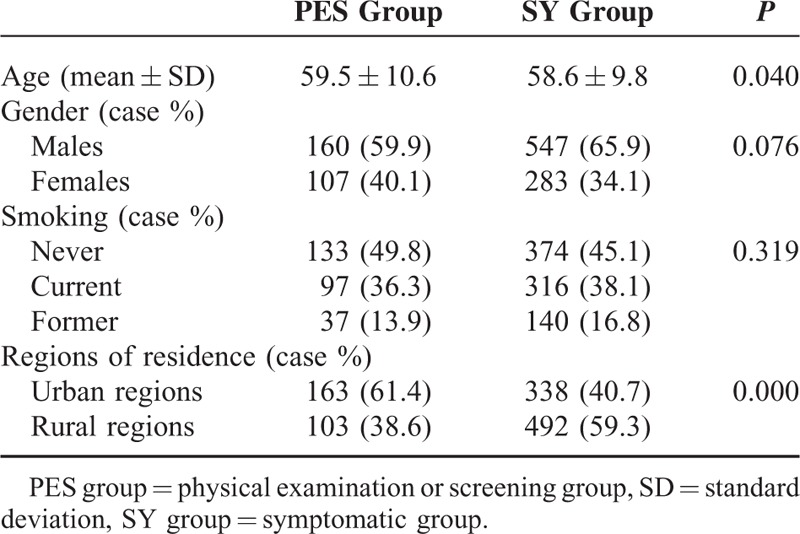
Baseline Characteristics

Of them, 24.3% (267/1097) patients were involved in the PES group and 75.4% (830/1097) in the SY group (Table 1).

The main clinical manifestations were cough (52.1%), expectoration (23.6%), blood in phlegm (15.9%), and pain (14.5%). The details of clinical manifestations are listed in Table 2.

**TABLE 2:**
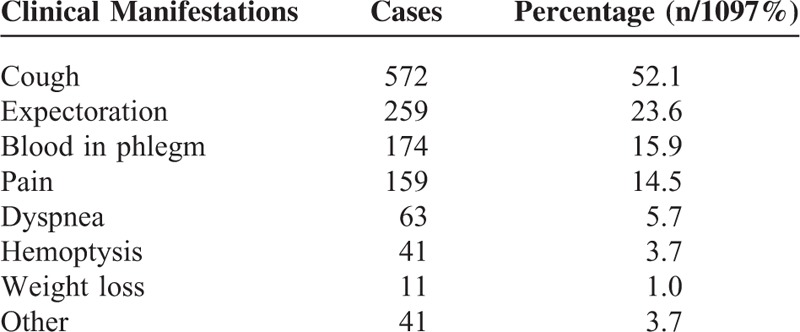
Details of Clinical Manifestations

Methods for lung cancer detection in the PES group were also analyzed. The main methods were low-dose multidetector computed tomography (LDCT) (110, 41.2%) and chest radiography (CR) (149, 55.8%). Other methods included biomarkers (3/267, 1.1%) and positron emission tomography (PET) (5/267, 1.9%). The 267 patients included 164 (61.4%) urban patients and 103 (38.6%) suburban or rural patients.

About 37.5% (100/267) patients in PES group spent <1 month from detection or first medical visit to diagnosis compared with 15.4% (128/830) patient in the SY group (*P* < 0.001). The percentage of patients who experienced multiple referrals or transfers in the PES group versus the SY group is lower in our unit, which is the largest tertiary referral center (120/267, 44.9% vs 680/830, 81.9%); also, a higher proportion of patients referred to our unit directly from a junior medical unit is found in PES group (43.8%, 117/267 vs 13.6%, 113/830) (*P* < 0.001). Details can be observed in Table 3.

**TABLE 3:**
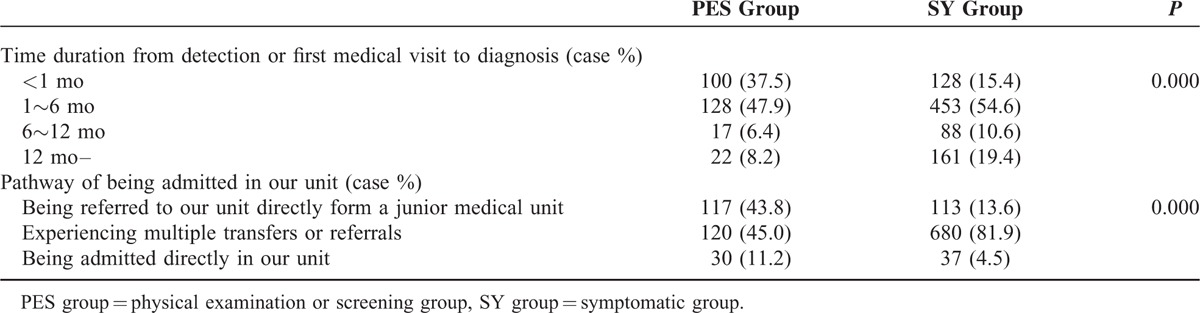
Process of Diagnosis and Admission

A significantly higher proportion of urban patients versus suburban/rural patients followed the new clinical mode (32.7%, 164/502 vs 17.3%, 103/595) (*P* < 0.001). A significantly higher proportion of urban patients at stage 0 or I versus the suburban/rural counterparts underwent the new clinical mode (62.8%, 108/172 vs 45.0%, 152/338) (*P* < 0.001).

Total in-hospital duration is significantly shorter in the PES group versus the SY group (15.6 ± 7.4 days vs 17.3 ± 8.5 days; *P* = 0.003). Details are specified in Table 4.

**TABLE 4:**
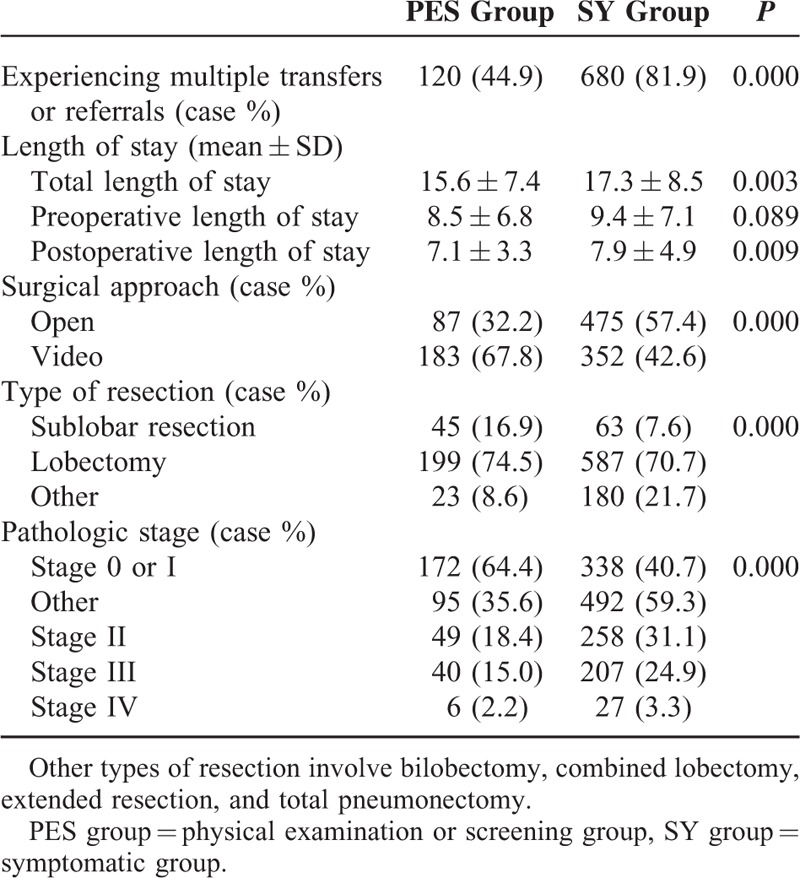
Clinical Characteristics in 2 Groups

Concerning surgical approaches, a significantly higher proportion in the PES group versus the SY group selected pulmonary VATS resection (67.8%, 183/267 vs 42.6%, 352/830) (*P* < 0.001). Regarding the type of resection, a significantly higher proportion in the PES group versus the SY group chose sublobar resection (16.9%, 45/267 vs 7.6%, 63/830) (*P* < 0.001) (Table 4).

The PES group has a significantly higher percentage of patients at stage 0 or I (172/267, 64.4%) than the SY group (338/830, 40.7%) (*P* < 0.001). Details are listed in Table 4.

Also, postoperative incidence of complications in 30 days is higher in the SY group versus the PES group (34.9% vs 27.3%, *P* = 0.022); however, the incidence rates of primary complications such as pneumonia or chest infection and aerodermectasia are not significantly different between the 2 groups (*P1* = 0.562, *P2* = 0.092). Details are listed in Table 5.

**TABLE 5:**
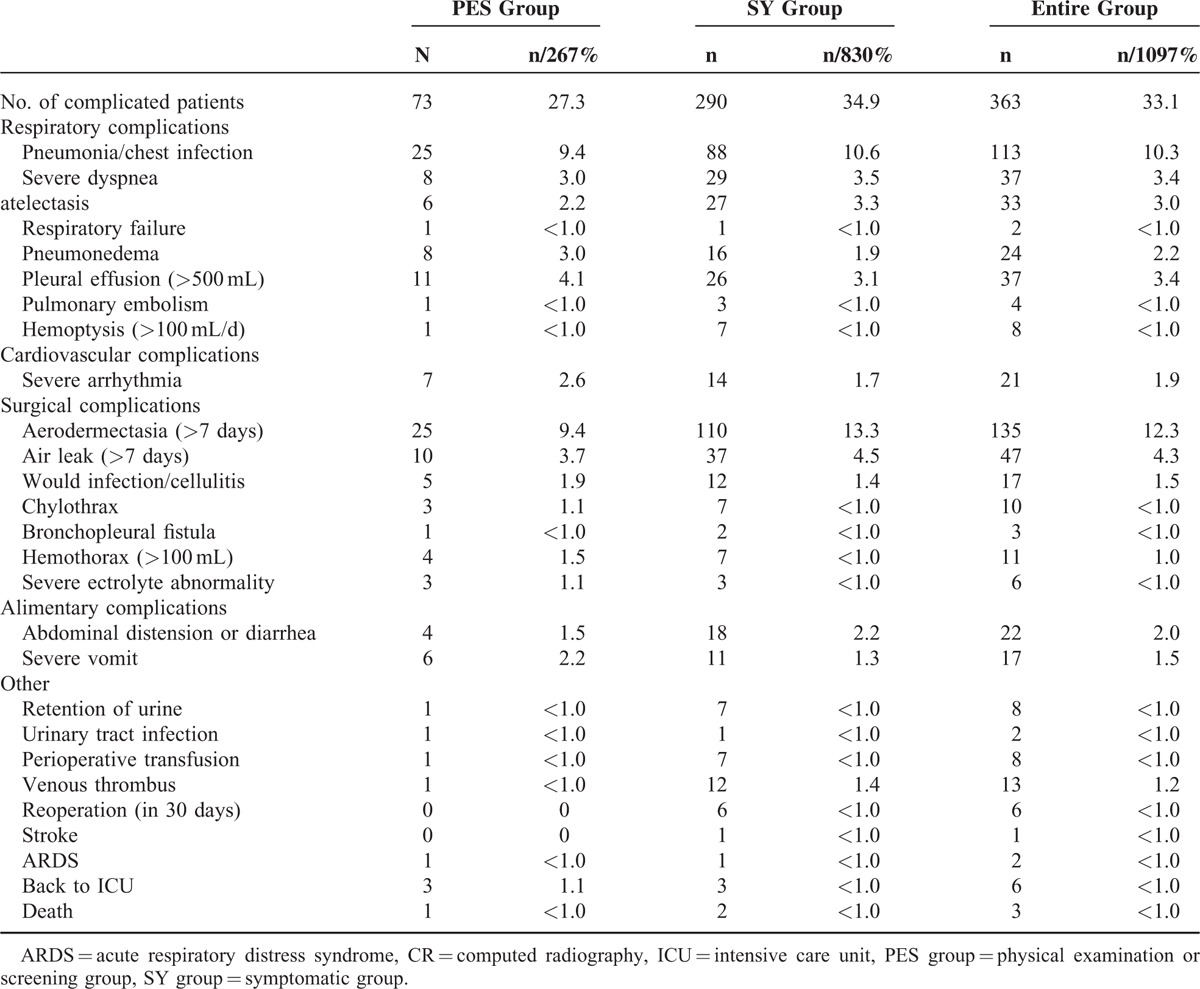
Complications in 2 Groups

## DISCUSSION

The expected survival rate of NSCLC patients is low, partly because the majority of patients are diagnosed at advanced stages when curative treatments are infeasible. Despite the successes from recent efforts in early detection and treatment of lung cancer, much work remains to be done. Compared with developed countries, the lung cancer patients in China have low 5-year survival rate, and not high quality of life, which could be summed up as “not long-living, not well-living.” The direct reason is the current triple-neglect situation, which is an attitude of neglecting prevention, diagnosis, and supportive care.^9,10^ Periodic PES with effective methods (eg, LDCT and CR) and strategies such as activities targeted at high-risk groups should be performed to promote public awareness or increase the possibility of detection of inchoate lung cancer. Although relevant administrations and medical practitioners persistently assert and stress the significance of periodic PES, the data and analyses are insufficient.

Chest LDCT, which has been investigated intensively in the last 2 decades, is considered to be more sensitive than CR for detection of early-stage lung cancers manifested as small, noncalcified, and solitary pulmonary nodules.^11,12^ Several large nonrandomized studies show that LDCT compared with CR could detect a larger number of lung cancers, most at an early and thus resectable stage. The spiral and multidetector CT scanners and LDCT screening for lung cancer have been widespread accepted in clinic, which facilitates the promotion of lung cancer screening services in China. Therefore, some lung cancer screening studies using LDCT have been conducted in China since the early 2000, though there are some limitations, such as lack of randomized controlled trial (RCT), small sample, single-center, and only urban patients. Moreover, the publicity of risk factors is insufficient and largely ignored, but it is not easy to obtain achievements with lack of resources and social support; thus, very little screening work has been performed.^9,10^ Among 267 patients undergoing detection of lung cancer via PES, the main method is CR (55.8%, 149/270), rather than LDCT (41.2%, 110/267). The use of LDCT so far is not as popular as we expected, which may be attributed to 4 reasons. First, no RCT on LDCT in China has been performed to confirm the capability of LDCT in reducing lung cancer specific mortality. In other words, we do not have Chinese-specific data or analysis, though enormous studies focus on LDCT. Second, the insufficiency of specific data and analysis, as well as other factors that might be inherently imbedded, may complicate the establishment of criteria for LDCT-based lung cancer detection. Third, the relatively high cost of LDCT has not been covered by national basic medical insurance. Fourth, rural patients prefer to use Chinese traditional medicine or chest x-ray examination only. This phenomenon has led more than 80% of lung cancer patients to miss the best treatment time, because it is too late for them to receive treatment by the time of identification.^9,10^ Our study finds that a significantly higher proportion of urban patients versus suburban/rural patients followed the new clinical mode, which may indicate that patients in city may be more likely to PES, than rural patients, though this speculation should be validated or confirmed via epidemiologically data.

For asymptomatic patients, especially those with risk factors, periodic PES is an initiative and effective way for early detection or observation of lung cancer. We believe that detecting more asymptomatic patients is propitious to diagnosis of early lung cancer. Meanwhile, patients in PES group underwent shorter time from detection or first medical visit to diagnosis, which to some extend means that more time and money may be saved. Moreover, the percentage of patients experiencing multiple transfers or referrals instead of passing a fast-track referral is lower in the PES group than in the SY group, which means the majority of the PES group was referred in our unit from a junior medical institution or admitted directly, a tertiary referral center in the province. This result also suggests that more time and money for multiple medical visits could be saved, which is better for early diagnosis and treatment of lung cancer. Nevertheless, more data and analysis are needed to further confirm that the prevalence of the new clinical mode can save the money and time for reduplicated medical visits. The results on lung cancer at the postoperative pathological stage show that a higher percentage of early-stage patients were found in the PES group. This might predict a better treatment effect in this group.

VATS resection offers advantages of alleviated postoperative pain, reduced loss of intraoperative blood, less operation time, better postoperative respiratory function, shorter in-hospital duration, and higher compliance with adjuvant chemotherapy, despite the controversy whether or not VATS resection outperforms open resection in terms of perioperative complications, mobility, and long-term prognosis.^13–20^ This study confirms that a higher percentage of patients in the PES group have undergone VATS lobectomy, than the SY group. This difference might be attributed primarily to the higher proportion of early-stage patients in the PES group, and also to other factors such as economy, and quality of education. Also, the higher proportion of early-stage patients in the PES group accounts for the higher use rate of sublobectomy, which may be more effective in preservation of lung function.^7,8^ Moreover, the use rate of sublobectomy or lobectomy is higher in the PES group. We do not exclude other factors or bias that may influence the conclusion in our study, but the new clinical mode leading to early diagnosis of lung cancer undoubtedly plays an important role.

Moreover, the postoperative incidence rate of complications in 30 days is lower in the PES group than in the SY group. This difference may be attributed to the higher proportion of early-stage lung cancer, higher use rate of VATS resection, and shorter in-hospital stay, and especially PES, though many factors may also influence the result.

In conclusion, patients following the new clinical mode are more likely to have VATS lobectomy with higher possibility to be detected at an early stage, and are also more likely to choose sublobectomy. All those actions may contribute to the treatment and prognosis. However, most of the 267 patients following the new clinical mode come from urban areas, compared with suburban or rural patients. Moreover, a higher proportion of urban patients at stage 0 or I were detected via PES compared with suburban or rural patients. These results suggest that the new clinical mode is more prevalent in urban patients. This fact requires relevant administrations and medical and health institutions to promote the propaganda of health awareness and significance of periodic PES, and encourage more people to care about personal health initiatively and effectively via periodic PES. The data, analytic findings, and conclusions in the present study may contribute to policy-making in clinic and public health, and will confirm the significance of periodic PES on a scientific basis.

To our best knowledge, this is the first study analyzing the association between clinical modes and clinical characteristics, though it is only a retrospective evaluation rather than a prospective randomized trial. We hope that future studies could confirm and stress the importance of periodic PES from the surgeons’ perspective and with more data and analysis. Nevertheless, some issues in our study need to be noticed and considered.

First, the patients were selected from a population surgically treated by a small group of surgeons in 1 unit between 2012 and 2013. The exclusion of some patients (details listed in the earlier parts) may confound the study, reduce its generalizability, and inevitably affect the results.

Second, our study is aimed at surgically treated patients in our unit.

Third, various methods with different availability and validity were used in early detection of lung cancer, but these methods were considered as a whole in the analysis to confirm the significance of PES.

Fourth, we approximately analyzed or evaluated the improvement disparity of periodic PES between urban and suburban/rural areas.

We tried to find some “tendency” or “clue” on the clinical characteristics and clinical modes, which could be an evidence to confirm the advantage of early detection and reflect the current situation of early detection (clinical mode). However, some unavoidable factors (eg, surgical approach and comorbidities) may confound our results. Further study is needed to discuss the relationships among those factors. Nevertheless, this “tendency” shows us the disparity between urban and rural patients on prevalence of early lung-cancer detection, reflecting the deficiency in publicity and education.

The government and society have placed insufficient values on prevention, especially early detection, resulting in a lack of emphasis on screening, early diagnosis, or supportive care. Thus, we do not have enough large sample or official data to support our conclusion. For example, it may be easily accepted that PES is more prevalent in urban cities than in rural areas, but no authoritative data are available to confirm this conclusion.

Far too little research has been done about the detection methods. In this study, we mainly discussed CR and LDCT, but not blood tumor biomarker analysis, endoscopy, or sputum cytology, as they are not commonly used for general detection in population. However, a comprehensive detection should be a multidisciplinary strategy. For example, blood-based markers should be developed to serve in conjunction with CT scans to reduce invasive workups and increase the cost-effectiveness of screening.

Moreover, future analyses involving more variables are needed to improve our study, which could better evaluate or refine our conclusions and economic analyses and may demonstrate the cost-effectiveness of scanning programs and physical examination.

In summary, significantly higher proportions of early lung cancer, use of VATS pulmonary resection, and sublobectomy as well as lower postoperative incidence of complications are found in patients with early tumor detection via PES. Therefore, more attention should be paid to the popularization and availability of PES.
